# RETRACTION: *Ganoderma lucidum* and *Auricularia polytricha* Mushrooms Protect against Carbofuran‐Induced Toxicity in Rats

**DOI:** 10.1155/ecam/9767181

**Published:** 2026-07-29

**Authors:** Evidence-Based Complementary and Alternative Medicine

RETRACTION: M. S. Hossen, M. M. Billah Prince, E. M. Tanvir, et al., “*Ganoderma lucidum* and *Auricularia polytricha* Mushrooms Protect against Carbofuran‐Induced Toxicity in Rats,” *Evidence-Based Complementary and Alternative Medicine*, 2018, 6254929, https://doi.org/10.1155/2018/6254929.

The above article, published online on 15 May 2018 in Wiley Online Library (https://wileyonlinelibrary.com), has been retracted by John Wiley & Sons Ltd. The retraction has been agreed due to concerns identified with the Figures [1]. Specifically:

• In Figure 6c (Positive control 2), there are unexpected repeated elements when compared to Figure 6a of [2].
• In Figure 6B (Positive control 1), there are unexpected, repeated elements when compared to:
  • Figure 6b of [2].
  • Figure 6a of [3].
  • Figure 3b, 3c and 3d of [4].
  • Figure 7b of [5].
3. In Figure 7, there are unexpected, repeated elements between panels b and c.
4. In Figure 7, there are unexpected, repeated elements between panels a and f, when stretched.
5. In Figure 7, there are unexpected repeated elements between panels b and e.
6. In Figure 7, there are unexpected repeated elements between panels c and e, when rotated 180°.
7. In Figure 7, panel f appears similar to panels a, b and g of [2], when resized.

Following assessment of the concerns, and in the absence of the original images from the authors in their response, the results and conclusions can no longer be considered reliable. The article is therefore retracted.

Sudip Paul agrees to the retraction, whilst no response was received by the remaining authors.
